# Supplementary material for: Guanabenz acetate, an antihypertensive drug repurposed as an inhibitor of Escherichia coli biofilm
Source: Microbiol Spectr. 2024 Sep 23;12(11):e00738-24. doi: 10.1128/spectrum.00738-24 (PMC11537090; doi:10.1128/spectrum.00738-24)
Supplement: Supplemental material — Table S1; Fig. S1 to S5. [file spectrum.00738-24-s0001.doc]

**Supplemental Data 1**

**Guanabenz acetate, an antihypertensive drug repurposed as an inhibitor of *Escherichia coli* biofilm**

Arakkaveettil Kabeer Farha,^a*^ Olivier Habimana,^a,b^ Harold Corke, ^a,b*^

^a^ Biotechnology and Food Engineering Program; and Key Laboratory of Science and Engineering for Health and Medicine of Guangdong Higher Education Institutes, Guangdong Technion-Israel Institute of Technology, Shantou, 515063, China

^b^ Faculty of Biotechnology and Food Engineering, Technion-Israel Institute of Technology, Haifa, 3200003, Israel

**Running Head: Guanabenz acetate inhibits *Escherichia coli* biofilm**

*Address correspondence to Harold Corke, [harold.corke@gtiit.edu.cn](mailto:harold.corke@gtiit.edu.cn)

Arakkaveettil Kabeer Farha, [farha.kabeer@gtiit.edu.cn](mailto:farha.kabeer@gtiit.edu.cn)

Table S1. List of genes affected by GABE treatment

| **Gene Name** | **Regulation** | **Product** | **Log2 fold**  **change** | ***p* value** |
| --- | --- | --- | --- | --- |
| *gadE* | Down | DNA-binding transcriptional activator GadE | -8.004 | <0.0001 |
| *hdeB* | Down | periplasmic acid stress chaperone HdeB | -7.613 | <0.0001 |
| *gadF* | Down | small regulatory RNA GadF | -7.130 | <0.0001 |
| *hdeA* | Down | periplasmic acid stress chaperone HdeA | -6.678 | <0.0001 |
| *arrS* | Down | small regulatory RNA ArrS | -6.137 | <0.0001 |
| *gadA* | Down | glutamate decarboxylase A | -6.076 | <0.0001 |
| *yhiD* | Down | inner membrane protein YhiD | -5.593 | <0.0001 |
| *gadB* | Down | glutamate decarboxylase B | -5.338 | <0.0001 |
| *prpE* | Down | propionyl-CoA synthetase | -5.078 | <0.0001 |
| *hdeD* | Down | acid-resistance membrane protein | -4.954 | <0.0001 |
| *prpD* | Down | 2-methylcitrate dehydratase | -4.904 | <0.0001 |
| *prpC* | Down | 2-methylcitrate synthase | -4.716 | <0.0001 |
| *prpB* | Down | 2-methylisocitrate lyase | -4.713 | <0.0001 |
| *gadC* | Down | L-glutamate:4-aminobutyrate antiporter | -4.091 | <0.0001 |
| *rseX* | Down | small regulatory RNA RseX | -4.047 | 0.008 |
| **Curli operon** | | | | |
| *csgB* | Down | curlin%2C minor subunit | -10.456 | <0.0001 |
| *csgA* | Down | curlin%2C major subunit | -8.984 | <0.0001 |
| *csgE* | Down | curli assembly component CsgE | -3.005 | 0.249 |
| *csgC* | Down | curlin chaperone | -2.961 | 0.444 |
| *csgD* | Down | DNA-binding transcriptional dual regulator CsgD | -2.569 | 0.241 |
| *csgF* | Down | curli assembly component CsgF | -2.454 | 0.289 |
| *csgG* | Down | curli secretion channel | -2.444 | 0.263 |
| **Cellulose operon** | | | | |
| *bcsB* | Down | cellulose synthase periplasmic subunit | -2.593 | 0.239 |
| *bcsZ* | Down | endo-1%2C4-D-glucanase | -2.070 | 0.255 |
| *bcsA* | Down | cellulose synthase catalytic subunit | -1.778 | 0.242 |
| *bcsC* | Down | cellulose synthase outer membrane channel | -1.432 | 0.177 |
| *bcsG* | Down | cellulose phosphoethanolamine transferase | -1.366 | 0.217 |
| *bcsE* | Down | c-di-GMP-binding protein BcsE | -1.154 | 0.194 |
| *dgcC* | Down | diguanylate cyclase DgcC | -1.291 | 0.241 |


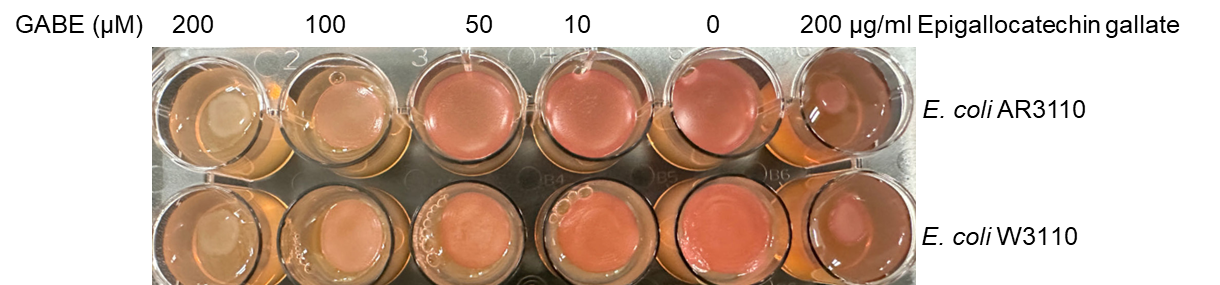


Figure S1. Congo red agar staining assay of *E. coli* strains. *E. coli* strains AR3110 and W3110 (2 µL) were spotted on LB salt-free agar supplemented with Congo red (40 µg/mL) and Coomassie brilliant blue (20 µg/mL) with varying concentration of GABE (0-200 µM) in a 24-well plate. The plate was incubated at 28°C for 96 h. Colony morphology was analyzed, and photographs were taken using the iPhone 14 Promax camera. Epigallocatechin gallate (EGCG) (200 µg/mL) was used as a positive control.


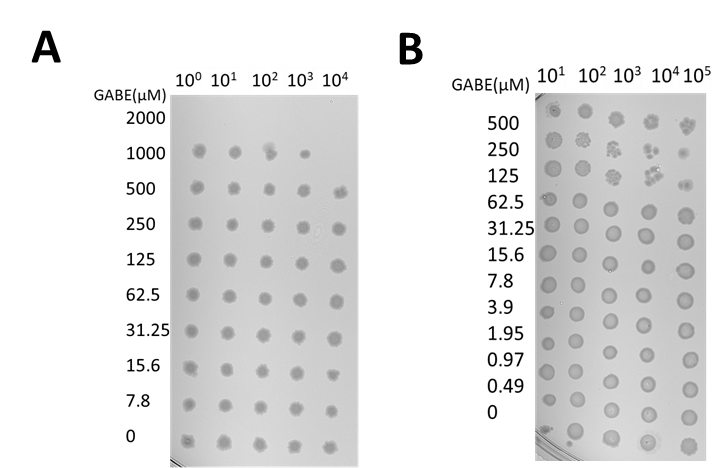


Figure S2. Spot assay analysis to confirm the viability of *E. coli* AR 3110 in liquid culture and in biofilm. (A) *E. coli* AR3110 was incubated in MH broth with various concentrations of GABE in a 96-well plate. After 24 h of incubation at 37°C, the bacterial culture from each well was serially diluted, and spotted on the LB agar plate, and the plate was incubated at 37°C for 24 h. (B) *E. coli* AR3110 was incubated in LB salt-free broth with various concentrations of GABE in a 96-well plate and incubated at 28°C for 48 h. At the end of incubation, the media were removed, biofilm was serially diluted and spotted on the LB agar plate, and the plate was incubated at 37°C for 24 h. The images were captured by a gel imaging instrument (Fusion-FX7.EDGE, Vilber).


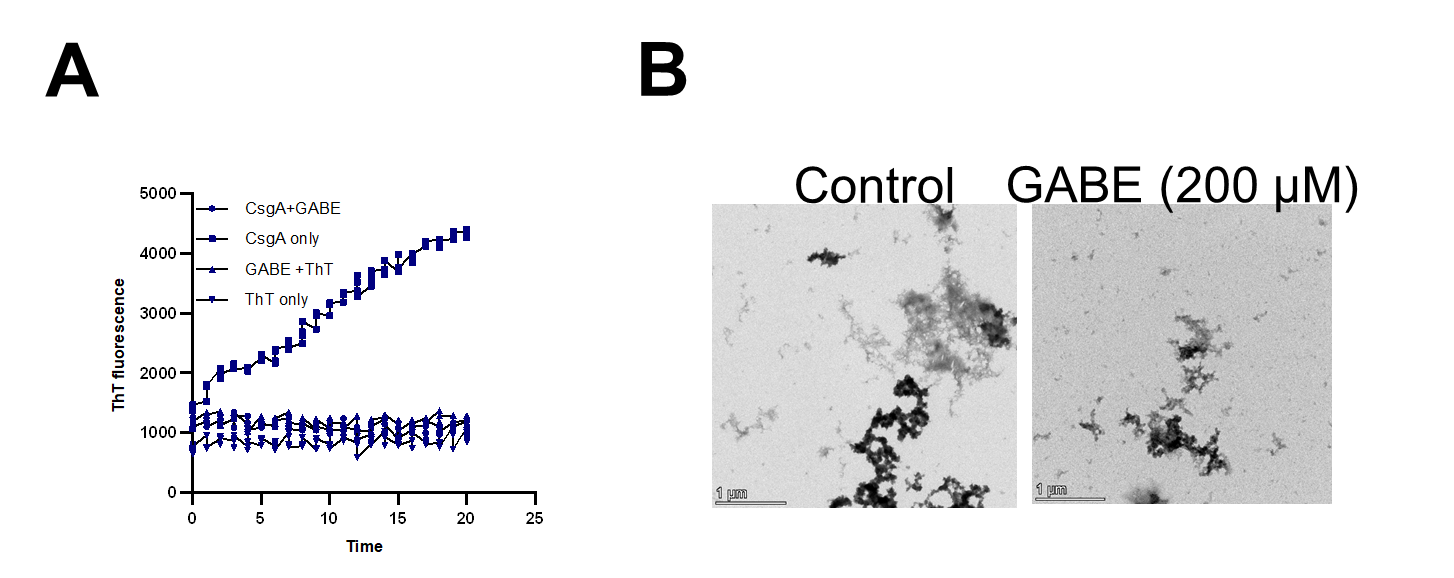


Figure S3. Effect of GABE on curli polymerization *in vitro*. (A) CsgA polymerization was monitored by thioflavin T (ThT) fluorescence over time with or without GABE. (B) TEM analysis of CsgA polymerization with or without GABE. Purified CsgA protein (Cusabio, Wuhan, China) was incubated with or without GABE in the presence of ThT and the fluorescence was measured (excitation/emission: 438/495) every 15 minutes for 20 h. The graph shows data from a single analysis. (B) TEM image of CsgA polymerization with or without GABE. CsgA was negatively stained with uranyl formate and observed by TEM.

\


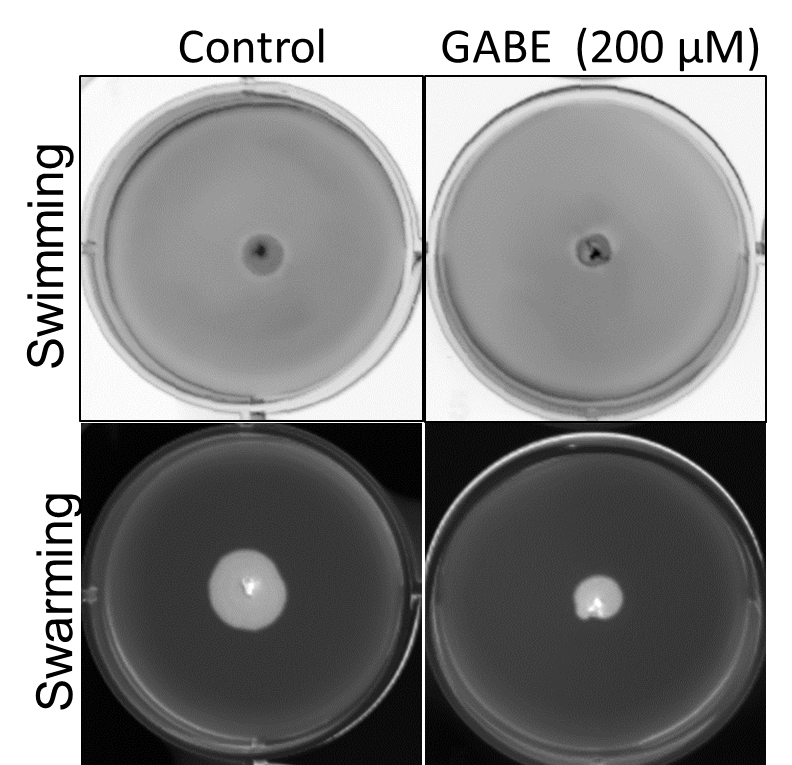


Figure S4. Effect of GABE on swimming and swarming motility of *E. coli* AR3110. E. *coli* AR3110 was spotted on swimming and swarming LB agar plates containing 0.3% and 0.5% agar in the presence or absence of GABE. All plates were incubated at 37°C for 24 h.


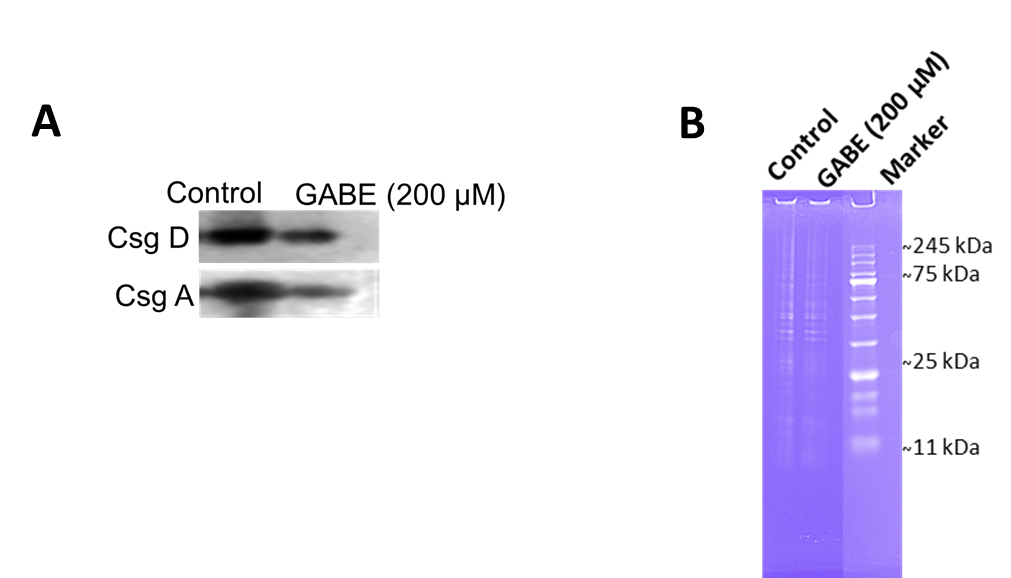


Figure S5. Effect of GABE on curli protein expression. (A) Western blot detection of expression of CsgA and CsgD proteins in *E. coli* AR3110 biofilm with or without GABE. (B). The SDS-PAGE profile of proteins extracted from *E. coli* AR3110 biofilm (gel imaging system, Fusion-FX7.EDGE, Vilber). *E. coli* AR3110 was grown on LB salt- free agar with or without GABE at 28°C for 48 h. After scrapping, the biofilms were treated with formic acid to depolymerize the curli subunits. Proteins were then separated using an SDS-polyacrylamide gel, and blots were probed with anti-CsgA and anti-CsgD antibodies.
